# Effect of material composition and thickness of orthodontic aligners on the transmission and distribution of forces: an in vitro study

**DOI:** 10.1007/s00784-024-05662-x

**Published:** 2024-04-19

**Authors:** Tarek M. Elshazly, Christoph Bourauel, Ahmed Ismail, Omar Ghoraba, Mostafa Aldesoki, Damiano Salvatori, Hanaa Elattar, Abdulaziz Alhotan, Yasmine Alkabani

**Affiliations:** 1 Oral Technology, Dental School, University Hospital Bonn, Welschnonnenstr. 17, Bonn, 53111 Germany; 2 Biomaterials Department, Faculty of Dentistry, Ain Shams University, Cairo, Egypt; 3 Institut Straumann AG, Basel, Switzerland; 4 Orthodontic Department, Faculty of Dentistry, Suez Canal University, Ismailia, Egypt; 5 Department of Dental Health, College of Applied Medical Sciences, King Saud University, Riyadh, Saudi Arabia; 6 Oral and Dental Research Institute, National Research Centre, Giza, Egypt

**Keywords:** Biomechanics, Orthodontic force, Tooth movement, Removable thermoplastic appliance, Pressure sensitive film, Stress analysis

## Abstract

**Objectives:**

To investigate the effects of material type and thickness on force generation and distribution by aligners.

**Materials and methods:**

Sixty aligners were divided into six groups (*n* = 10): one group with a thickness of 0.89 mm using Zendura Viva (Multi-layer), four groups with a thickness of 0.75 mm using Zendura FLX (Multi-layer), CA Pro (Multi-layer), Zendura (Single-layer), and Duran (Single-layer) sheets, and one group with a thickness of 0.50 mm using Duran sheets. Force measurements were conducted using Fuji® pressure-sensitive films.

**Results:**

The lowest force values, both active and passive, were recorded for the multi-layered sheets: CA Pro (83.1 N, 50.5 N), Zendura FLX (88.9 N, 60.7 N), and Zendura Viva (92.5 N, 68.5 N). Conversely, the highest values were recorded for the single-layered sheets: Duran (131.9 N, 71.8 N) and Zendura (149.7 N, 89.8 N). The highest force was recorded at the middle third of the aligner, followed by the incisal third, and then the cervical third. The net force between the incisal and cervical thirds (F_I_-F_C_) showed insignificant difference across different materials. However, when comparing the incisal and middle thirds, the net force (F_I_-F_M_) was higher with single-layered materials. Both overall force and net force (F_I_-F_M_) were significantly higher with 0.75 mm compared to those with a thickness of 0.50 mm.

**Conclusions:**

Multi-layered aligner materials exert lower forces compared to their single-layered counterparts. Additionally, increased thickness in aligners results in enhanced retention and greater force generation. For effective bodily tooth movement, thicker and single-layered rigid materials are preferred.

**Clinical relevance:**

This research provides valuable insights into the biomechanics of orthodontic aligners, which could have significant clinical implications for orthodontists. Orthodontists might use this information to more effectively tailor aligner treatments, considering the specific tooth movement required for each individual patient. In light of these findings, an exchangeable protocol for aligner treatment is suggested, which however needs to be proven clinically. This protocol proposes alternating between multi-layered and single-layered materials within the same treatment phase. This strategy is suggested to optimize treatment outcomes, particularly when planning for a bodily tooth movement.

## Introduction

In the era of digital and aesthetic dentistry, orthodontic aligners have increasingly become a preferred choice over traditional braces, thanks to their transparency and comfort [1]. Additionally, clear aligners are often the treatment of choice for adults who are more prone to periodontitis and gingivitis [2]. They are custom-made, removable splints, typically produced through the thermoforming process of plastic sheets. Each aligner in the treatment series is specifically designed to achieve a tooth’s translation of 0.2 mm or a rotation of about 2° over 1 to 2 weeks [3–5]. These sheets can be made of either conventional polymers [6, 7] or, more recently, smart polymers [8, 9].

The force system of aligners is complex, with multiple factors influencing the treatment outcomes [10]. The biomechanical behavior of aligners is determined by factors such as their material properties [11, 12], thickness [13–15], adaptation to teeth [16], extension and trimming design [17–21], and the amount of activation [15, 22]. Despite ongoing advancements in the field of aligners, discrepancies still exist between the planned virtual outcomes and the actual clinical results [3, 6]. The force system of aligners has been extensively studied using various methods, including numerical finite element method [15, 23–25], together with experimental methods such as force sensors [26], pressure-sensitive films [18, 27–29], and customized biomechanical systems [21, 30–32].

Pressure-sensitive films, unlike standard sensors, have a reduced thickness, minimizing interference during testing with aligners and requiring less relief. This results in more user-friendly testing conditions. These films can directly measure the pressure between two surfaces, offering a rapid, straightforward, and dry measurement method [18]. Nevertheless, they are sensitive to technique and are significantly influenced by temperature, light, humidity, and load-rate [33]. They are available in two forms (mono-sheet and two-sheet), each is supplied with a range of sensitivity options. The two-sheet form comprises an A-transfer film and a C-developer film, with a combined thickness of about 100 μm. The A-film contains microcapsules that rupture under pressure, releasing chemicals that react with an active layer on the C-film. The resulting change in optical density, from pale pink to dark red, indicates the pressure intensity [34]. A specialized scanner can digitize these films, converting them into numerical and visual data [18].

Pressure-sensitive films were initially introduced in orthodontics to assess the occlusal changes after the end of treatment [35–37], however, Barbagallo et al. [28] were among the first to employ these films for measuring the force exerted by orthodontic aligners. More recently, Cervinara et al. [27] employed them to analyze the force distribution applied by aligners to the upper central incisors. Elshazly et al. [18] investigated the impact of aligner trimming line design on force levels and distribution using the same technique. Additionally, Zamani et al. [29] measured forces generated by both passive and active aligners, especially with different types of attachments.

Despite the growing number of research on orthodontic aligners, the scientific literature on their biomechanical behavior still lacks comprehensive data. Many factors affect the resulting movement, including root shape and length and the point of contact between aligner and tooth. Pure translations are hardly achieved with clear aligners, because by their nature it is not possible to accurately predict the point of force application and control the root. Therefore, the aim of the current study is to shed light on how aligner material composition and thickness might affect the magnitude and distribution of forces on the facial surface of a maxillary central incisor when a facio-lingual bodily movement was planned. To this end, we have employed pressure-sensitive films, a methodology that, to our knowledge, has not been previously explored in this context.

## Materials and methods

Pressure-sensitive films (Fuji® Prescale Film; Fuji Film, Tokyo, Japan) were used to measure the forces exerted by orthodontic aligners made of various materials. The optimal parameters applied in this study were based on a sensitivity analysis reported in a recent study [18]. A digital model of the upper dental arch, including all sixteen teeth (Digimation Corp., St Rose, Louisiana, USA) of a dentulous maxilla, was generated and imported as a stereolithography (STL) file into an advanced 3D image processing and editing software (3-matic 16.0; Materialise, Leuven, Belgium). This model was digitally redesigned using the software tools, resulting in three district models, each serving a different purpose in the current study.

**Fig. 1:**
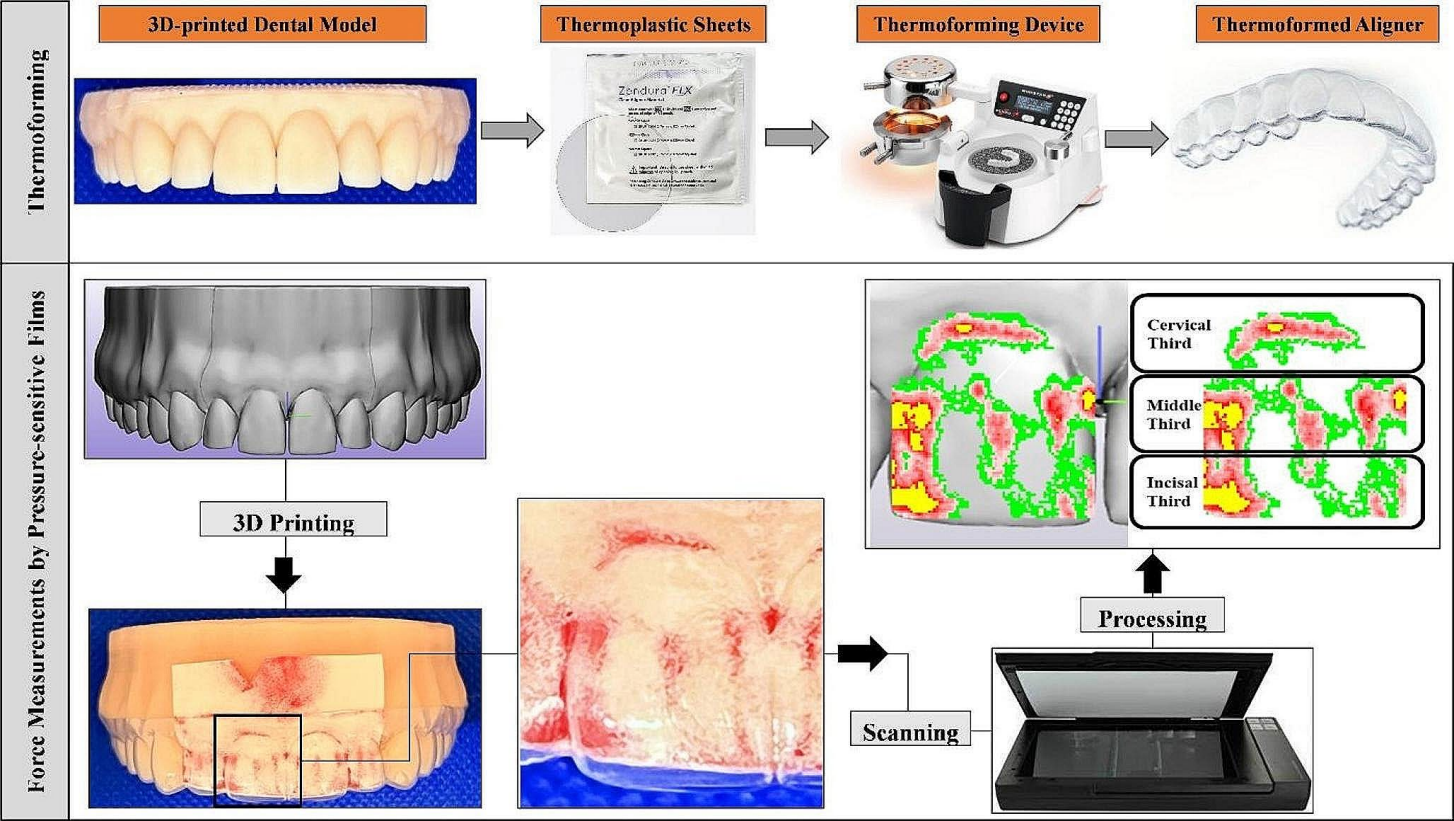
A schematic draw showing the workflow followed in the current study for thermoforming and force measurements. Fuji^®^ Pressure-sensitive films were employed to measure the normal contact force on the facial surface of an upper right central incisor. The force measurements were taken at three distinct levels: Cervical (C), Middle (M), and Incisal (I)

The first model was tailored for the thermoforming process. It was designed as an aligned model with the teeth in their correct positions, free of malposition. The model height was measured 15 mm from the occlusal table to the model base. Additionally, a shallow depression was intentionally designed 2 mm away from the gingival margin of the teeth to ensure consistent and standardized aligner trimming (Fig. 1). The second model, also an aligned design, included a created 100 μm space around the four incisors and their corresponding gingival areas (housing space). This space was introduced to offset the inherent thickness of the pressure-sensitive films, which are 100 μm thick according to the data provided by the manufacturer (Fig. 1). The second model was used for passive force measurements, which indicate the aligner’s retention by measuring the force exerted between the aligned teeth and the aligner. In the third model, the right central incisor (Tooth 11) was bodily translated 0.2 mm facially, and the 100 μm housing space was also included. This model was designed for active force measurements, evaluating the force exerted between the malaligned tooth and the aligner. These models were printed with a resin (P pro resin; Straumann, Basel, Switzerland) using a digital light processing (DLP) 3D printer (P20+; Straumann, Basel, Switzerland), whose minimum accuracy is 34 μm.

**Fig. 2:**
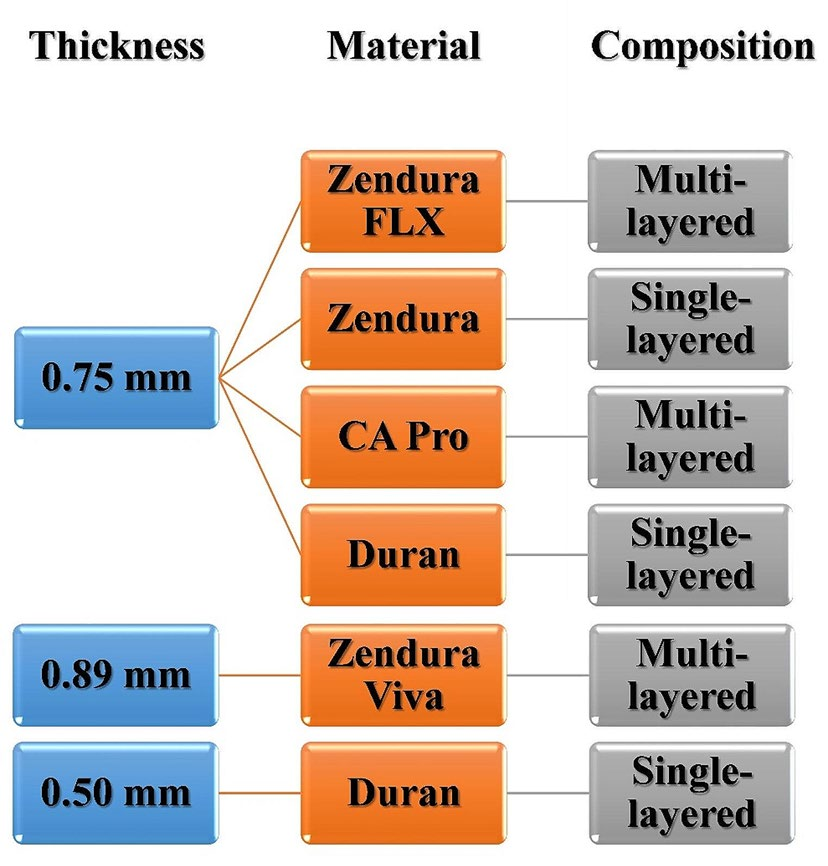
A scheme showing the classification of the current study groups (*n* = 10); based on aligner thickness and material composition

Five thermoforming materials were used (as detailed in Table 1): Zendura FLX, Zendura Viva, CA Pro, Zendura, and Duran. All aligners were fabricated from sheets of the same 0.75 mm thickness (except Zendura Viva at 0.89 mm) and were trimmed to a straight 2 mm-extended trimming line design (*n* = 10) (Fig. 1). Additionally, to investigate the effects of aligner thickness, a further group (*n* = 10) of Duran sheets with a thickness of 0.50 mm was produced for comparison with the 0.75 mm thickness aligners made from the same material. In total, 60 aligners were fabricated (Fig. 2).

The thermoforming process was conducted according to the manufacturer’s specifications for each material and thickness (Table 1), using a thermoforming device (Biostar; Scheu-dental GmbH, Iserlohn, Germany). The 3D-printed model was consistently centered on the device platform for each fabrication to minimize inhomogeneity when deep drawing the sheets over the model. All aligners were produced by a single trained technician using the same 3D-printed models.

**Table 1:** Data on aligner sheets used in the current study (Manufacturers’ data)

Name	Manufacturer	Thickness/ Thermoforming Code	Thermoforming conditions	Material composition
Duran^®^	Scheu-Dental (Iserlohn, Germany)	0.75 mm / Code (112)	Heating for 25 s at 220 °C, and pressure-forming at 4.8 bar, then cooling for 60 s	Single-layered sheet of Polyethylene terephthalate glycol-modified (PETG)
		0.50 mm / Code (101)	Heating for 20 s at 220 °C, and pressure-forming at 4.8 bar, then cooling for 20 s	
CA^®^ Pro	Scheu-Dental (Iserlohn, Germany)	0.75 mm / Code (112)	Heating for 25 s at 220 °C, and pressure-forming at 4.8 bar, then cooling for 60 s	Three-layer sheet of a soft thermoplastic elastomeric layer between two hard layers of co-polyester
Zendura FLX™	Bay Materials (Fremont, USA)	0.75 mm / Code (162)	Heating for 50 s at 220 °C, and pressure-forming at 5.8 bar, then cooling for 60 s	Three-layer sheet of a soft thermoplastic elastomeric layer between two hard layers of co-polyester
Zendura Viva™	Bay Materials (Fremont, USA)	0.89 mm / Code (162)	Heating for 50 s at 220 °C, and pressure-forming at 5.8 bar, then cooling for 60 s	Three-layer sheet of a soft thermoplastic elastomeric layer between two hard layers of co-polyester
Zendura™	Bay Materials (Fremont, USA)	0.75 mm / Code (162)	Heating for 50 s at 220 °C, and pressure-forming at 5.8 bar, then cooling for 60 s	Single-layered sheet of thermoplastic polyutherane (TPU).

In this study, the two-sheet type of pressure-sensitive film was utilized for force measurements. Each aligner was tested for both passive and active forces. To record active forces (between the malaligned third printed model and the adapted aligner), the low-pressure (LW) films with a measuring range of 2.5–10.0 MPa were used. For recording the passive forces (between the aligned second printed model and the adapted aligner), super-low-pressure (LLW) films, with a measuring range of 0.5–2.5 MPa were employed.

The films were precisely cut and seated with extreme caution to fit the designed housing space. Aligners were seated with extreme caution to prevent inadvertent pressure during the measurements. A single, well-trained operator conducted all the measurements. Room humidity and temperature were monitored using an indoor digital climate station (Technoline WS 9440 Indoor climate station; Technotrade GmbH, Wildau, Germany) during scanning. The recorded values for humidity and temperature (35 ± 4% and 25 ± 3 °C, respectively) were input into the scanning software to be factored into the measurement calculations.

Each pressurized film was scanned using a special scanner (EPSON perfection V300 series; SEIKO Epson CORPORATION, Japan) with a resolution of 0.125 (200 dpi), as recommended by the manufacturer 1. The force was digitalized and quantified using coupled software (FPD-8010E analysis system; Fuji® Film, Tokyo, Japan). This software enables the direct calculation of force values, which is determined by multiplying the average pressure (MPa) by the pressurized area (mm^2^), as per the software manual.

Force = Pressure × area

Force values (N) at the three thirds [Incisal (I), Middle (M), Cervical (C)] of the facial surface of (Tooth 11), as well as the overall force (N) over the facial surface of the tooth were recorded (Fig. 1). Additionally, for each aligner, the passive force was subtracted from the active force, and this resultant value (absolute force) was reported. The absolute force might reflect how the material responds to deformation.

**Fig. 3:**
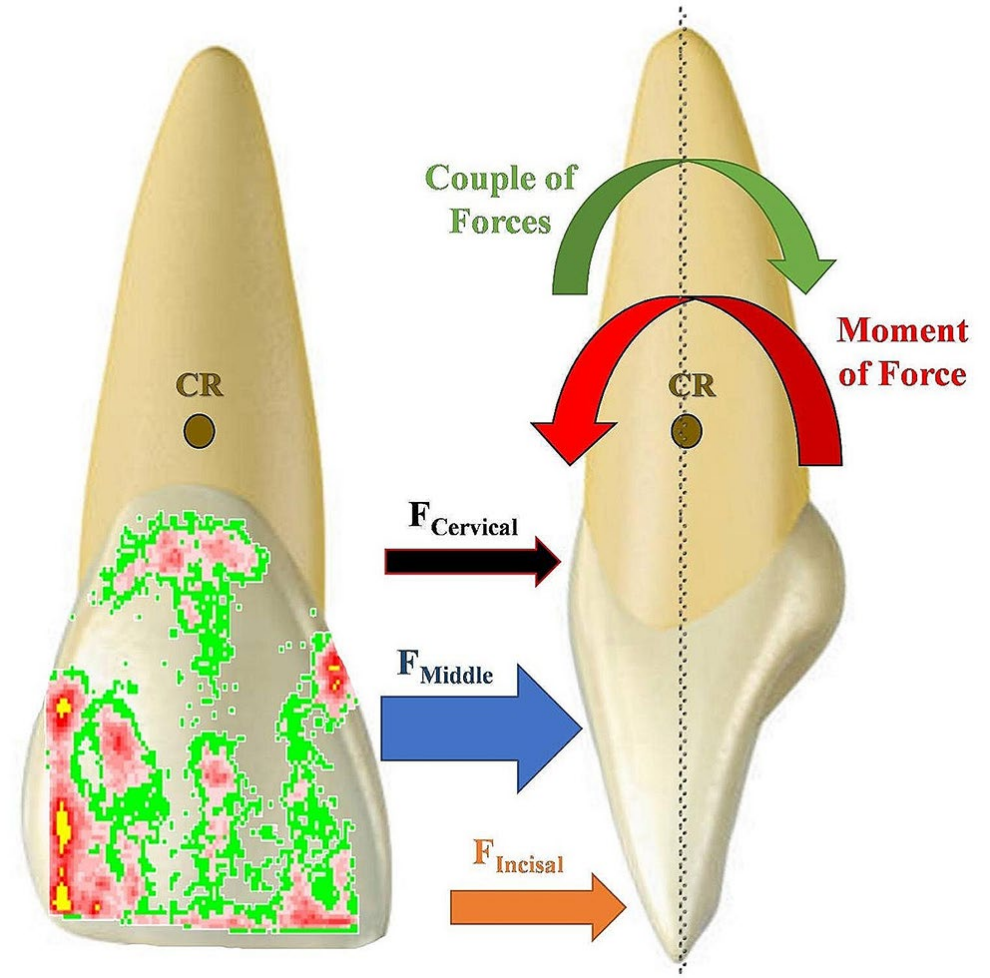
A schematic explanation of the moment of force system exerted by an orthodontic aligner on an upper central incisor. F_I_ is applied at a distance from the center of resistance (CR), creating a tipping moment of force that tends to tip the incisor. To counteract this tipping moment, forces are applied closer to the CR, such as F_C_ and F_M_, generating a couple of forces. The higher the net force (F_I_-F_C_ or F_I_-F_M_) results in a greater couple of forces, reducing the tendency for tipping

Moreover, the distribution of forces (F_C_, F_M_, and F_I_) relative to the center of resistance (CR) is crucial in determining whether the orthodontic aligner treatment results in tipping or generates a couple of forces to promote bodily movement [6]. Therefore, the net force (F_I_-F_C_) between the active forces at the incisal third (I) and cervical third (C), as well as the net force (F_I_-F_M_) between the active forces at the incisal third (I) and the middle third (M) were calculated for each group. This was done to investigate the effect of the material and thickness on the moment of force, and consequently, on the type of tooth movement (Fig. 3).

### Statistical analysis

Numerical force values were presented as mean ± standard deviation. Normality was assessed by examining data distribution and applying the Shapiro-Wilk test. As the data showed a parametric distribution, they were analyzed using one-way ANOVA followed by a post-hoc Tukey HSD Test. The significance level (α) was set at *p* ≤ 0.05. Statistical analysis was performed using IBM SPSS Statistics version 25 for Windows (IBM, Endicott, NY, USA).

## Results

**Fig. 4:**
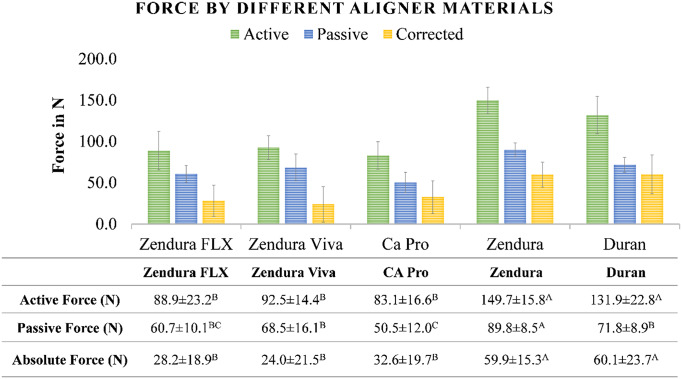
Active, passive, and absolute forces (in N) transmitted to the facial surface of the upper central incisor by orthodontic aligners made of different thermoformed materials, different uppercase superscript letters indicate a statistically significant difference within the same horizontal row, *significant (*p* < 0.05)

The active force values generated across the entire facial surface of tooth 11 ranged from 83.1 to 149.7 N. Passive force values varied from 50.5 to 89.8 N, while the absolute force values spanned from 24.0 to 60.1 N. The highest force values were observed in the single-layered materials (Duran and Zendura), with no significant differences between them. Conversely, the lowest force values were noted in the multi-layered materials (CA Pro, Zendura FLX, and Zendura Viva), with no significant differences among them in terms of active and absolute forces (Fig. 4).

**Fig. 5:**
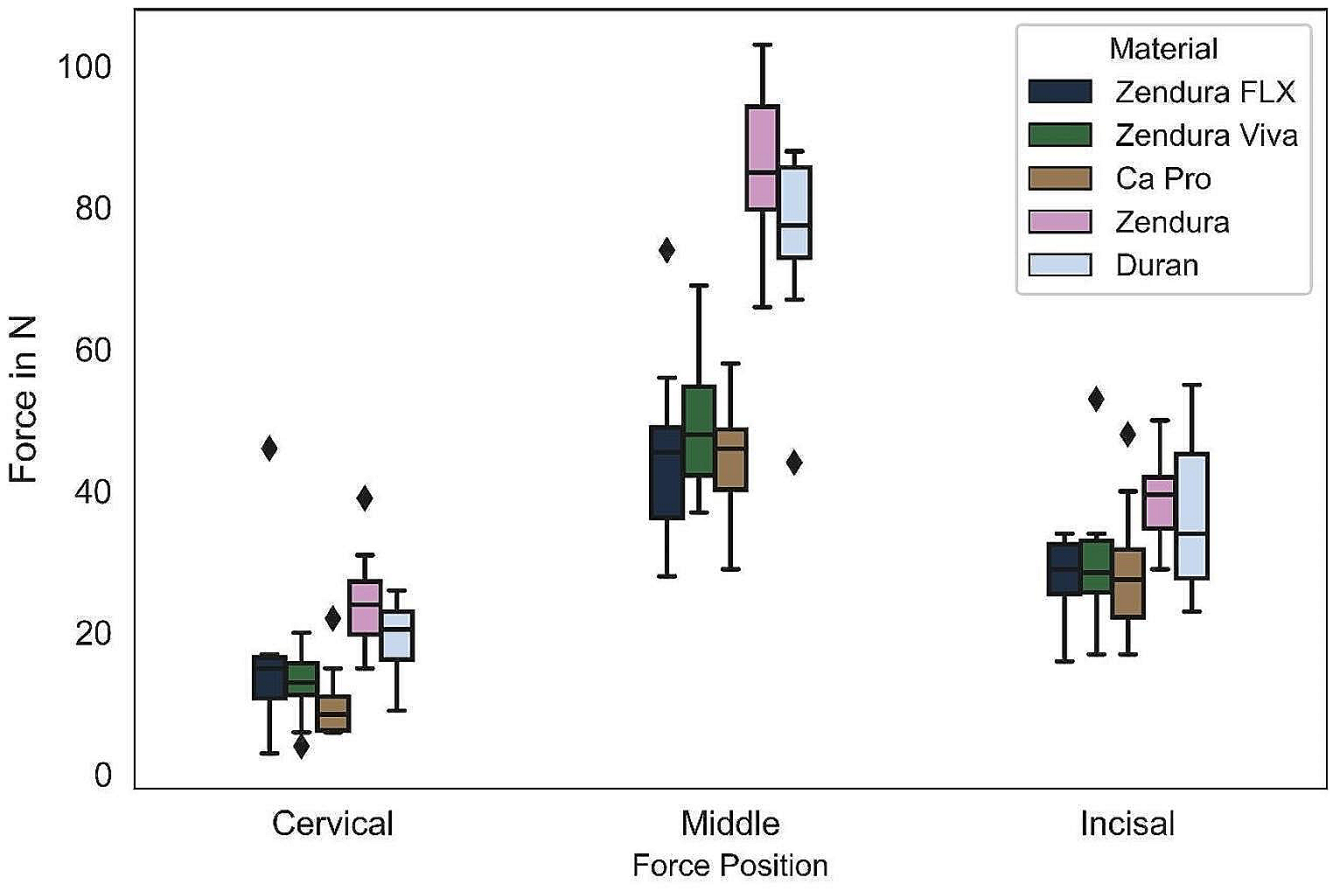
A box plot chart depicting the active forces (measured in N) applied to the facial surface of an upper central incisor by orthodontic aligners fabricated from various thermoformed materials. The forces were measured at three distinct positions along the facial surface: Cervical (C), Middle (M), and Incisal (I)

The distribution of forces across the facial surface of Tooth 11 was uneven, with concentrations of force at specific positions (Figs. 1, 4 and 5). The force varied significantly at different positions on the tooth, with some areas experiencing substantially higher forces than others. Dividing the tooth surface into three equal thirds revealed that the active force at the middle third (F_M_) was significantly higher than that at the incisal (F_I_) and cervical thirds (F_C_). Statistical analysis showed no significant difference in the net active force (F_I_-F_C_) among different materials. However, when comparing the net active force (F_I_-F_M_), single-layered materials exhibited higher net forces than multi-layered materials (Table 2).

Furthermore, aligners made from Duran material with a thickness of 0.75 mm displayed higher forces than those with a thickness of 0.5 mm. There was no significant difference in the net active force (F_I_-F_C_) between the two thicknesses. However, the net active force (F_I_-F_M_) was higher in the thicker aligners (Table 3).

**Table 2:** Active forces (in N) exerted on the facial surface of a maxillary central incisor by aligners made of different thermoformed materials. Forces were recorded at the three thirds of the facial surface: namely Cervical (C), Middle (M), Incisal (I), as well as the net active forces (F_I_-F_C_) between the incisal (I) and the cervical thirds (C) and the net active forces (F_I_-F_M_) between the incisal (I) and the middle thirds (M)

Tooth third	Active Force (N) (Mean ± SD)					*p-value*
	Zendura-FLX	Zendura-Viva	CA Pro	Zendura	Duran	
C	16.0 ± 11.4^ABb^	12.7 ± 4.9^Bc^	10.1 ± 5.1^Bc^	24.4 ± 7.1^Ac^	19.0 ± 5.7^ABc^	0.008*
M	45.4 ± 13.3^Ba^	50.0 ± 11.0^Ba^	44.4 ± 8.0^Ba^	85.9 ± 12.1^Aa^	75.7 ± 13.1^Aa^	< 0.01*
I	27.5 ± 6.3^Bb^	29.8 ± 9.8^ABb^	28.6 ± 9.7^ABb^	39.4 ± 6.6^Ab^	37.2 ± 11.5^ABb^	< 0.01*
*p-value*	< 0.01*	< 0.01*	< 0.01*	< 0.01*	< 0.01*	
F_I_-F_C_	11.5^A^	17.1^A^	18.5^A^	15.0^A^	18.2^A^	0.899
F_I_-F_M_	-17.9^B^	-20.2^B^	-15.8^B^	-46.5^A^	-38.5^A^	< 0.01*

Different uppercase lowercase superscript letters indicate a statistically significant difference within the same horizontal row and vertical column respectively, *significant (*p* < 0.05)

**Table 3:** Active, passive, and absolute forces (in N) exerted on the facial surface of a maxillary central incisor by two different thicknesses of aligners made of Duran sheets. Forces were recorded at the three thirds of the facial surface: namely Cervical (C), Middle (M), Incisal (I), as well as the net active forces (F_I_-F_C_) between the incisal (I) and the cervical thirds (C) and the net active forces (F_I_-F_M_) between the incisal (I) and the middle thirds (M)

	Force in N (Mean ± SD)		p-value
	0.5	0.75	
Active	86.6 ± 16.2^B^	131.9 ± 22.8^A^	*p* < 0.01*
Passive	57.0 ± 7.9^B^	71.8 ± 8.9^A^	*p* < 0.01*
Absolute	29.6 ± 14.9^B^	60.1 ± 23.7^A^	*p* < 0.01*
Tooth third	Active Force in N (Mean ± SD)		**p-value**
	**0.5**	**0.75**	
C	9.4 ± 4.2^Bc^	19.0 ± 5.7^Ac^	*p* < 0.01*
M	45.5 ± 10.0^Ba^	75.7 ± 13.1^Aa^	*p* < 0.01*
I	31.7 ± 8.3^Ab^	37.2 ± 11.5^Ab^	*p* < 0.01*
p-value	*p* < 0.01*	*p* < 0.01*	
F_I_-F_C_	22.3^A^	18.2^A^	0.308
F_I_-F_M_	-13.8^B^	-38.5^A^	

Different uppercase lowercase superscript letters indicate a statistically significant difference within the same horizontal row and vertical column respectively, *significant (*p* < 0.05)

## Discussion

Understanding the biomechanical system of orthodontic aligners is crucial for achieving efficient and successful treatment outcomes. Investigating the material properties of clear aligners provides clinicians with insights into their limitations [38]. The incorporation of innovative materials with enhanced properties has the potential to significantly improve the efficacy of orthodontic aligner treatments [8, 9]. However, most aligner suppliers still rely on conventional thermoforming techniques. This method can introduce variations in material properties [7] and lead to geometric inaccuracies [18], affecting both the fit of the aligner and the forces transmitted to the teeth. Thus, a comprehensive study is needed to understand how the type and thickness of thermoplastic materials influence the biomechanics of aligners. Such investigations are crucial for controlling the forces exerted and achieving more predictable outcomes in tooth movement. To our knowledge, the influence of these factors has yet to be thoroughly explored, particularly using the pressure-sensitive films.

The absence of periodontal ligament (PDL) representation in orthodontic experimental studies often results in higher forces than the physiological orthodontic limit (0.1–1.2 N) [11]. The force values obtained in this study (83.1–149.7 N) are significantly higher than those reported in the literature. This disparity may be attributed to the innovative methodology employed, which includes the use of pressure-sensitive films and a specialized scanner equipped with software capable of directly quantifying force values. However, these pressure-sensitive films have limitations, as they provide localized one-dimensional force measurements on a single tooth surface. In contrast, clear aligners exert three-dimensional forces simultaneously across multiple tooth positions and directional axes, resulting in a lower overall resultant force. This discrepancy represents a limitation of the current research approach. In addition, the geometrical deviations in the manufacturing process of the model (3D Printing), the aligner (thermoforming), and the pressure film itself may play a significant role in the determination of the forces. While the offset of 100 μm was designed to compensate for the film thickness, these variations may add up and affect the actual contact between the aligner and the teeth, resulting in a shift toward higher forces. Another factor is that the dry conditions and room temperature reduce the stiffness of the aligner material compared to the intraoral conditions.

Utilizing pressure-sensitive films, Barbagallo et al. [28] reported force values of approximately 28.0 N in an in vivo study. Similarly, Cervinara et al. [27] reported values in pressure units (around 3.3 MPa), not in forces units. Although measured in different units, our force values are comparable to Cervinara’s findings, considering the average measured pressurized surface area in our experiment was about 40 mm^2^, and the force (N) is calculated as the product of pressure (MPa) and area (mm^2^).

Thermoplastic materials used in aligners exhibit viscoelastic behavior, meaning they inherently possess elastic and plastic characteristics. Consequently, when an aligner splint is deformed over the targeted tooth, these materials tend to partially return to their original shape. This inherent property is the fundamental basis for the generation of forces by orthodontic aligners employed for tooth movement [6]. The effectiveness of tooth movement with orthodontic aligners is determined by the force systems they generate, including both forces and moments. The ratio of moments to forces acting on the tooth can vary significantly and influence the type of tooth movement [39]. In removable orthodontic appliances, forces are typically applied to the crown of the tooth, away from the center of resistance (CR) in the root, creating a moment leading to tooth tipping. The magnitude of this moment is directly proportional to the applied force and the distance between the force application point and the CR. Consequently, altering the location of force application impacts the type of moment generated and, hence, the type of tooth movement. Specifically, as the applied force moves closer to the CR, the resulting tipping motion diminishes while the tendency for bodily translation of the tooth increases [6, 40]. Thus, the distribution and application point of the force on the tooth surface significantly affect the type of tooth movement, determining whether it is bodily or tipping. A bodily movement requires the application of a higher force at the cervical region than the incisal [21].

Consistent with previous studies [18, 27], the thermoformed orthodontic aligners in the current study exhibited close and intimate contact with the teeth, leading to the generation of passive forces. The higher levels of passive forces observed with the Zendura single-layered sheets suggest superior initial retention compared to some multi-layered sheets, like CA Pro and Zendura Viva. Similarly, the higher passive force generated with thicker Duran aligners indicates greater retention. A previous review by Upadhyay and Arqub [6] highlighted that intimate contact between aligners and teeth should facilitate a more uniform distribution of stress across the tooth’s surface. However, the tooth’s surface contour also plays a key role in determining how forces are distributed [15]. Consequently, uneven contact between the aligner and tooth surface was observed, resulting in areas of relief and others of close contact.

In line with the research by Lombardo et al. [41], our study found that the single-layered sheets (Zendura and Duran) showed higher absolute forces, indicating higher rigidity, while the multi-layered sheets (Zendura Viva, Zendura FLX, and CA Pro) exhibited lower absolute forces, indicating higher flexibility. Additionally, the active forces exerted by single-layered materials were higher than those generated by the multi-layered materials.

Moreover, the strength of forces varied across different positions of tooth surface, with the middle third consistently exhibiting the highest force intensity, followed by the incisal and then the cervical third, in agreement with prior studies [14, 17, 20]. This force distribution pattern makes achieving bodily tooth movement challenging and endorses more crown tipping. Rossini et al. [42] concluded similar findings in a systematic review. The net forces between the incisal and cervical thirds exhibited comparable values among different materials. However, the net forces between the incisal and middle thirds were higher with single-layered materials. This observation suggests an advantage in using rigid single-layered material for planning bodily tooth movement. It suggests an interchangeable protocol for aligner treatment, to be validated clinically, involving the use of flexible multi-layered material followed by rigid single-layered material within each treatment step. It should be noted that the results reflect the clinical situation at the beginning of the treatment step. In a clinical setting, the tooth is free to move in the palatal direction within the limits of the periodontal ligament. Thus, a new contact between aligner and tooth in the palatal surface will be established that will affect the total force system. Nevertheless, the results suggest that more rigid aligners tend to maintain a more uniform contact along the tooth length, thus are less prone to cause tipping movements when a bodily movement is planned.

The thickness of the aligner plays a crucial biomechanical role [20]. Variations in material thickness lead to changes in force-deformation characteristics, aligner stiffness, and the forces transmitted [14, 20]. Additionally, increased thickness results in greater local and overall deformations of the aligner, reducing tooth-to-aligner contact areas and, consequently, aligner retention [16]. This is consistent with the findings of the current study. The cervical region of a thermoformed aligner is particularly important as it experiences the most significant thinning during thermoforming [18]. As a result, it is more prone to deformation and irregular forces generation [17, 18]. The present study found that thicker aligners generated significantly higher active and passive forces compared to thinner ones, aligning with previous research [13, 22]. Moreover, the net active force (F_I_-F_M_) was higher with thicker aligner, promoting bodily tooth movement.

Several limitations of this in vitro study should be acknowledged:

1. The technique-sensitivity of pressure films might lead to a wide variability in the measured force.
2. Extra forces were unavoidably transmitted during the insertion and removal of the aligner, particularly in the presence of undercuts, existing by default in both passive and active aligners.
3. The scanning analysis produced a two-dimensional image of a three-dimensional surface.
4. The effect of the PDL and bone, which significantly contribute to the stress distribution in the orthodontic appliances, was not included.
5. The precision of the aligners was affected by inherent variability introduced during the thermoforming and trimming. To minimize this variability, aligner fabrication via thermoforming strictly followed the manufacturer’s guidelines, using the same printed model for consistency. Additionally, a standardized guided straight trimming line design, consistently positioned 2 mm apical to the gingival line, was uniformly implemented for all aligners. This approach aimed to prevent additional finishing and polishing steps, which could negatively impact the mechanical properties of the aligner at the gingival region [17, 18]. Furthermore, the sample size was increased to ten aligners per group (*n* = 10) to enhance the statistical robustness of the study and account for residual variability.

Future research could apply the same methodology can be applied to explore various types of tooth movements. The suggested protocol alternating softer and more rigid aligners during each treatment step should be proven clinically. Additionally, there is potential to investigate the influence of attachments, including their shapes and positions, on force distribution and tooth movement dynamics. Moreover, integrating of 3D printing technology into aligner manufacturing is a promising path for investigation. This technology offers enhanced control over aligner design and thickness. Hence, this method could also facilitate an in-depth examination of the advantages of 3D-printed aligners compared to traditional thermoformed aligners.

## Conclusion

Within the limitation of the current in vitro study the following observations can be drawn:

1. When orthodontic aligners are used, the forces are not uniformly distributed across the tooth surface. Instead, they concentrate on specific repeatable areas, which can be identified as force application areas.
2. The force distribution pattern on the tooth surface induced by aligners tends to favor tipping movements.
3. The aligner material type notably influences the force generation when aligner splint deformation occurs over a malaligned tooth. Compared to the single-layered sheets of Zendura and Duran, the three-layer sheets of Zendura Viva, Zendura FLX sheets and CA-Pro can generate lower initial forces to the teeth.
4. The rigid single-layered aligner materials may promote bodily tooth movement, more than the flexible multi-layered aligner materials.
5. An exchangeable protocol for aligner treatment is suggested, which however needs to be proven clinically. This protocol involves using the flexible multi-layered material followed by the rigid single-layered material within each treatment step. This approach may optimize the aligner treatment outcomes, especially when a bodily movement of teeth is planned.

## Data Availability

No datasets were generated or analysed during the current study.

## Footnotes

1 https://fujiprescalefilm.com/fpd.php.

## Declarations

### Research highlights

•Force distribution transferred to the tooth by aligners achieves a tipping motion.

•Single-layered aligner materials promote bodily tooth movement.

•Multi-layered aligner materials are more flexible and apply less force.

•A thicker aligner is more retentive and exerts more force to support bodily movement.

•An interchangeable protocol (Flexible/Rigid materials per each step) is advisable.
